# Association between pelvic bone marrow dosimetry and acute hematologic toxicity during concurrent chemoradiotherapy for gynecologic malignancies

**DOI:** 10.1093/jrr/rraf084

**Published:** 2026-01-15

**Authors:** Chengliang Zhou, Jie Chen

**Affiliations:** Department of Radiation Oncology, The Fourth Affiliated Hospital of Soochow University, 9 Chongwen Road, Suzhou Industrial Park, Suzhou City, Jiangsu 215100, P.R. China; Department of Radiation Oncology, The Fourth Affiliated Hospital of Soochow University, 9 Chongwen Road, Suzhou Industrial Park, Suzhou City, Jiangsu 215100, P.R. China

**Keywords:** gynecologic malignancies, radiotherapy, chemotherapy, bone marrow dosimetry, acute hematologic toxicity

## Abstract

Pelvic radiotherapy for gynecologic malignancies damages the primary active bone marrow reservoir, inducing hematologic toxicity exacerbated by chemotherapy. Optimizing pelvic bone marrow dose–volume constraints is critical to mitigate myelosuppression and maintain treatment efficacy. The present retrospective cohort study analyzed patients with gynecological cancer (*n* = 61) undergoing concurrent chemoradiotherapy between August 2021 and August 2024. Associations between pelvic bone marrow (PBM) dose–volume parameters and acute hematologic toxicity (AHT) were systematically evaluated. All patients received intensity-modulated radiotherapy encompassing pelvic lymph node regions, with weekly complete blood count monitoring during and for 2 weeks after treatment. The overall incidence of AHT was 70.5% (43/61), with grade ≥ 2 and ≥ 3 AHT occurring in 63.9% (39/61) and 30.0% (14/61) of patients, respectively. Multivariate analysis identified PBM-V_15_ as an independent predictor of grade ≥ 2 AHT [odds ratio (OR), 2.653; 95% CI, 1.054–6.682; *P* = 0.038], with an optimal cutoff threshold of 80.44% [area under the curve (AUC), 0.854]. Notably, a lower PBM (LPBM)-V_5_ specifically predicted grade ≥ 3 AHT (OR, 1.425; 95% CI, 1.022–1.987; *P* = 0.037), with a threshold of 91.25% (AUC, 0.695). Implementing bone marrow-sparing strategies by restricting PBM-V_15_ to <80.44% significantly reduced the grade ≥ 2 AHT risk, while a stringent LPBM-V_5_ constraint (< 91.25%) was pivotal for preventing severe (grade ≥ 3) AHT. These dose–volume parameters should be incorporated into optimization protocols for pelvic radiotherapy in gynecological malignancies.

## INTRODUCTION

Gynecological malignancies remain a leading cause of cancer-related mortality among women worldwide, with cervical and endometrial cancers accounting for the majority of cases. According to the World Health Organization (WHO), cervical cancer alone caused >342 000 deaths globally in 2020, while the incidence of endometrial cancer, a gynecological tumor, is increasing [1]. Although multimodal therapies combining surgery, radiotherapy and chemotherapy have improved the outcomes of patients with locally advanced disease, population-based studies have indicated that most patients still require extensive pelvic radiotherapy to achieve locoregional control [2, 3]. However, the therapeutic benefits of radiotherapy are offset by radiation-induced toxicity to the pelvic bone marrow (PBM), a critical hematopoietic reservoir. This toxicity is exacerbated in concurrent chemoradiotherapy (CCRT) regimens, where synergistic effects between radiation and systemic chemotherapeutic agents result in grade ≥ 3 acute hematologic toxicity (AHT) in 20–50% of patients, jeopardizing treatment continuity and survival outcomes [4, 5].

The target area for pelvic radiotherapy typically includes the tumor itself as well as the surrounding pelvic lymph node region. Due to the anatomical proximity of these tumors and the frequent involvement of large pelvic lymph nodes, extensive pelvic irradiation is often necessary [6, 7]. However, radiation-induced bone marrow damage is an unavoidable side effect, particularly in large-volume radiation therapy, where substantial portions of the pelvic region are irradiated, resulting in increased radiation doses to the bone marrow, which may lead to bone marrow suppression.

The adult PBM accounts for 40% of the body’s active hematopoietic tissue of the body [8], serving a pivotal role in peripheral blood cell production. Its hematopoietic function is influenced by various factors, including age and concurrent treatments [9, 10]. Although CCRT increases the hematologic toxicity risk, its survival benefits justify continued clinical application [11]. Among these variables, the radiation dose to the pelvic bone marrow has emerged as one of the few key variables that can be actively controlled through radiation therapy planning optimization.

The pathophysiology of radiation-induced myelosuppression involves multifaceted mechanisms. Specifically, ionizing radiation can disrupt mitochondrial serine catabolism in hematopoietic stem cells, causing ferroptosis due to reduced nicotinamide adenine dinucleotide phosphate hydride (NADPH) [12]. Nynrin preserves hematopoietic stem cell function by inhibiting mitochondrial permeability transition pore (mPTP) opening, thus reducing reactive oxygen species (ROS) accumulation [13]. Radiation-induced bystander effects damage human hematopoietic stem cells through oxidative stress, leading to DNA damage responses, cell cycle arrest and p53-dependent apoptosis [14]. Furthermore, pathways such as the p53-Puma upregulated modulator of apoptosis, granulocyte colony-stimulating factor/Stat3/basic leucine zipper ATF-like transcription factor and ROS-p38 pathways are implicated in promoting apoptosis, differentiation and senescence of hematopoietic stem cells, as well as damaging their niche [15]. An *in vitro* study has demonstrated a dose-dependent reduction in both the quantity and colony formation capacity of murine bone marrow–derived hematopoietic stem/progenitor cells (HSPCs) following radiation exposure. Early-stage HSPCs may exhibit heightened vulnerability to ionizing radiation [16]. Both high and low doses of radiation can affect hematopoietic stem cells; however, the mechanisms differ. High-dose radiation mainly causes DNA damage and activates repair and apoptotic pathways [17], while low-dose radiation is more associated with oxidative stress, epigenetic changes and metabolic alterations [18].

Several studies have shown that the pelvic irradiation dose as well as radiotherapy techniques are closely related to the occurrence of bone marrow suppression, although these results are slightly different [19–23]. A meta-analysis incorporating 65 clinical studies reported comparable findings regarding dose thresholds, with variations observed in specific parameters while maintaining consistent overall ranges. The radiation exposure parameters were specifically summarized as follows: V_10_ < 86–90%, V_20_ < 65–80%, V_40_ < 18.4–41% and V_50_ < 9–10% [24].

Optimizing the pelvic radiation dose and minimizing bone marrow suppression are crucial for improving treatment outcomes and the quality of life in patients with pelvic malignancies. In this context, exploring strategies to optimize the pelvic radiation dose and mitigate bone marrow suppression holds significant clinical and research value.

## MATERIALS AND METHODS

### Patients

The present study included a retrospective cohort of postoperative cervical and endometrial cancer patients who underwent CCRT without brachytherapy at the Department of Radiation Oncology, The Fourth Affiliated Hospital of Soochow University (Suzhou, China) between August 2021 and August 2024. All cases were staged according to International Federation of Gynecology and Obstetrics (FIGO) guidelines based on postoperative pathology: cervical cancer per FIGO 2018 [25] and endometrial cancer per FIGO 2023 [26].

#### Inclusion criteria

The inclusion criteria were as follows: (i) histopathologically confirmed cervical or endometrial malignancies; (ii) age between 18 and 75 years, with a performance status of 0–1; (iii) no contraindications for radiotherapy and chemotherapy; (iv) indication for pelvic radiotherapy, with target volumes encompassing the pelvic lymph node region; (v) completion of the planned pelvic radiotherapy regimen; and (vi) routine weekly complete blood count (CBC) monitoring during radiotherapy and for 2 weeks post-treatment.

#### Exclusion criteria

The exclusion criteria were as follows: (i) history of prior pelvic radiotherapy; (ii) radiotherapy target volume excluding the pelvic lymph node region; (iii) incomplete radiotherapy regimen; (iv) history of hematologic disorders; (v) diagnosis of multiple primary malignancies; (vi) pregnancy or lactation; and (vii) concurrent enrollment in other clinical trials.

### Radiotherapy workflow

Immobilization and simulation. Patients were positioned supine with both arms raised and forearms crossed, resting on the forehead. A vacuum cushion was used for immobilization. Treatment planning was based on contrast-enhanced CT scans (3 mm slice thickness) covering the region from the T12 vertebral body to the mid-femur. Patients with contrast allergies underwent non-contrast CT scans only.

Target delineation. The simulation CT images were imported into the treatment planning system for delineation of target volumes and organs at risk (OARs). Target volumes were contoured according to standard radiotherapy guidelines for cervical and endometrial cancer [6, 7]. Delineation was initially performed by an attending physician and subsequently reviewed by a senior physician (associate chief physician or higher).

Gross tumor volume (GTV). The GTV included tumors, comprising the primary tumor (GTV_p_) and metastatic lymph nodes (GTV_nd_).

Clinical target volume (CTV). The CTV included the GTV and the pelvic lymph node region.

Planning target volume (PTV). The PTV was generated by expanding the GTV and CTV with a 0.8–1.0 cm margin to account for setup uncertainties and organ motion.

OARs. The OARs included the rectum, bladder, femoral head, spinal cord, bowel and colon*.*

Dose prescription. The dose constraints for the target volumes were as follows: At least 95% of the PTV should receive ≥100% of the prescribed dose; <5% of the PTV should receive <95% of the prescribed dose; and <5% of the PTV should receive >105% of the prescribed dose.

The dose constraints for OARs were as follows: Rectum, V_45_ < 65% and D_mean_ < 35 Gy; bladder, V_40_ < 60% and D_2cc_ < 65 Gy; femoral heads, D_max_ <50 Gy and V_30_ < 50%; spinal cord, D_max_ < 45 Gy; bowel, D_max_ < 55 Gy and V_40_ < 30%; and colon, D_max_ < 55 Gy and V_45_ < 35%.

All patients underwent intensity-modulated radiotherapy (IMRT) using seven to nine fields. Seventeen patients received a local boost to the metastatic lymph nodes; among these, three also received an additional local boost to the primary tumor site due to positive resection margins. The median radiation dose for the pelvic lymph node region was 45 Gy (range: 45–50.4 Gy), that for the GTV_p_ was 55 Gy (range: 50–60 Gy) and that for the GTV_nd_ was 55 Gy (range: 50–60.4 Gy). Plan evaluation included dose–volume histogram analysis, conformity index, homogeneity index and target coverage.

### Pelvic bone marrow delineation

The PBM was contoured on bone window CT images and classified into three subregions: (i) lumbosacral bone marrow (LSBM), extending from the upper border of L5 to the lower border of the sacrum; (ii) iliac bone marrow (IBM), encompassing the iliac crest and extending superiorly to the femoral heads; and (iii) lower PBM (LPBM), comprising the ischium, acetabulum and proximal femur, ranging from the upper border of the femoral heads to the lower border of the ischial tuberosities. The volume of each region receiving 5–45 Gy (in 5 Gy increments), and the mean dose were quantified.

### Chemotherapy

Chemotherapy consisted of weekly cisplatin (40 mg/m^2^; intravenous infusion over 60 min) administered concurrently with pelvic radiotherapy. Cisplatin was withheld under the following conditions: white blood cell count <2.0 × 10^9^/L, absolute neutrophil count <1.0 × 10^9^/L, platelet count <50 × 10^9^/L or creatinine clearance <50 ml/min.

### Hematologic toxicity

CBCs were monitored from the first day of radiotherapy and at least once per week during radiotherapy and for 2 weeks post-radiotherapy. AHT (including leukopenia, neutropenia, anemia and thrombocytopenia) was graded according to the Common Terminology Criteria for Adverse Events (CTCAE) version 5.0, published by the U.S. National Cancer Institute (NCI) in 2017 [27]. The highest observed toxicity grade was recorded as the final toxicity assessment.

### Statistical analysis

All statistical analyses were performed using SPSS version 28.0 (IBM Corp.). Continuous variables are presented as the mean ± SD and were compared between groups using an unpaired *t*-test. Categorical variables are presented as frequencies (*n*) and percentages (%), and were analyzed using the χ^2^ test.

Univariate analysis was conducted using independent *t*-tests. Following the TRIPOD guidelines [28], we performed a multivariable logistic regression to identify independent predictors of AHT. To minimize the omission of potential confounders, variables with a *P*-value <0.20 from the univariate analysis were included as candidates. For endpoints with a limited number of events (Grade ≥ 3 AHT), the model was simplified to prevent overfitting by selecting representative dose–volume histogram (DVH) parameters. Based on DVH plot separation, V_5_, V_10_, V_15_ and V_20_ were chosen, but V_30_ and V_35_ were excluded due to collinearity despite *P* < 0.05 in univariate analysis.

Receiver operating characteristic (ROC) curve analysis was performed for significant variables identified in logistic regression analysis to determine optimal threshold values. The area under the curve (AUC), sensitivity and specificity were calculated to assess predictive performance. The Youden Index was further applied to refine the threshold selection, balancing sensitivity and specificity.

*P* < 0.05 was considered to indicate a statistically significant difference for all analyses.

## RESULTS

### Patients and tumor characteristics

**Fig. 1:**
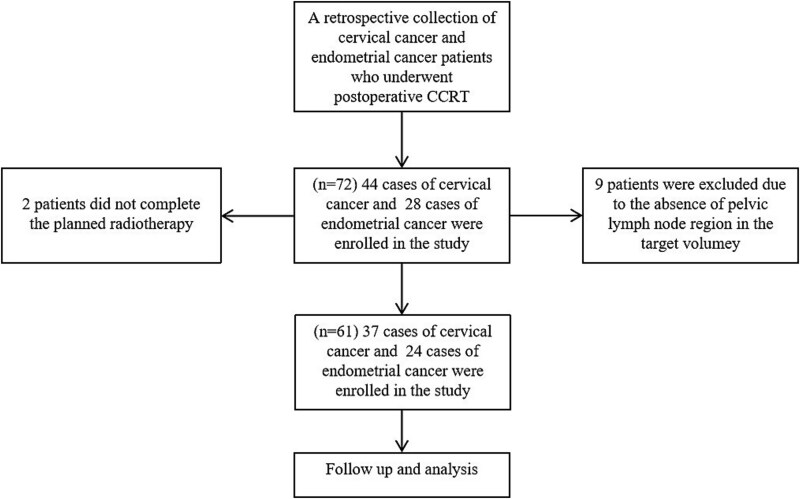
Study flowchart. Abbreviation: CCRT, concurrent chemoradiotherapy.

As shown in Fig. 1, a total of 72 patients were included in the present study. However, 11 patients did not meet the inclusion criteria and were excluded. Among them, 9 patients were excluded due to the absence of the pelvic lymph node region in the target volume and 2 patients did not complete the planned radiotherapy. Ultimately, 61 patients were included in the final analysis. Baseline patient characteristics are summarized in Table 1. The mean age of the patients was 58 years (range, 27–75 years). Among those aged >60 years (*n* = 27), 18 experienced grade ≥ 1 AHT, 18 experienced grade ≥ 2 AHT and 6 experienced grade ≥ 3 AHT. Among those aged ≤60 years (*n* = 34), 25 experienced grade ≥ 1 AHT, 21 experienced grade ≥ 2 AHT and 8 experienced grade ≥ 3 AHT. There was no significant difference in the incidence of AHT between the age groups. Among 37 cases of cervical cancer (FIGO 2018 staging), stage III was predominant (18 cases). Of 24 endometrial cancer (FIGO 2023 staging) cases, stage II was most frequent (9 cases). All patients received weekly cisplatin-based chemotherapy, with 8 patients completing 6 cycles, 32 patients completing 5 cycles and 21 patients completing 4 cycles. Pre-treatment hematological parameters were as follows: the mean hemoglobin level was 126.3 g/L (12.6 g/dL; range, 110–165 g/L [11.0–16.5 g/dL]), the mean white blood cell count was 6.1 × 10^9^/L (range, 4.02–11.81), the mean absolute neutrophil count was 4.4 × 10^9^/L (range, 2.29–9.42) and the mean platelet count was 207.5 × 10^9^/L (range, 112–382).

**Table 1:** Patients and tumor characteristics

Variable	Count	Value
Total patients	61	100%
Age		
>60 years	27	44.3%
≥1 AHT	18	29.5%
≥2 AHT	18	29.5%
≥3 AHT	6	9.8%
≤60 years	34	55.7%
≥1 AHT	25	41.0%
≥2 AHT	21	34.4%
≥3 AHT	8	13.1%
Stage (FIGO)		
Cervical cancer	37	60.7%
I	13	21.3%
II	4	6.6%
III	18	29.5%
IV	2	3.3%
Endometrial cancer	24	39.3%
I	7	11.5%
II	9	14.7%
III	8	13.1%
IV	0	0%
Chemotherapy		
Cisplatin (6 cycles)	8	13.1%
Cisplatin (5 cycles)	32	52.5%
Cisplatin (4 cycles)	21	34.4%
Pre-treatment hematology		
Hemoglobin (g/L)		126.3 (110–165) [12.6 (11.0–16.5) g/dL]
White blood cell count (×10^9^/L)		6.1 (4.02–11.81)
Absolute neutrophil count (×10^9^/L)		4.4 (2.29–9.42)
Platelet count (×10^9^/L)		207.5 (112–382)

Abbreviations: AHT, acute hematologic toxicity; FIGO, International Federation of Gynecology and Obstetrics.

Staging criteria: Cervical cancer per FIGO 2018, endometrial cancer per FIGO 2023.

Note: The FIGO stage was based on postoperative pathological findings. The included Stage IVA cases were upstaged from a lower clinical stage after primary surgery.

### Hematologic toxicity

As shown in Table 2, a total of 43 patients experienced AHT, with leukopenia and neutropenia being the most frequently observed adverse events. The numbers of grade 1 adverse events were as follows: leukopenia, 5 cases; neutropenia, 6 cases; anemia, 7 cases; and thrombocytopenia, 2 cases. For grade 2 adverse events, the corresponding numbers were 24, 12, 3 and 7 cases. Grade 3 events occurred in 12 (leukopenia), 8 (neutropenia), 2 (anemia) and 4 (thrombocytopenia) patients. Grade 4 events included 2 cases of leukopenia, 2 cases of neutropenia and 1 case of thrombocytopenia, with no grade 4 anemia cases reported.

**Table 2:** Acute hematologic toxicity

Toxicity	Grade 0	Grade 1	Grade 2	Grade 3	Grade 4
AHT	18	4	25	12	2
Leukopenia	18	5	24	12	2
Neutropenia	33	6	12	8	2
Anemia	49	7	3	2	0
Thrombocytopenia	47	2	7	4	1

Note: AHT represents the composite highest grade among leukopenia, neutropenia, anemia and thrombocytopenia for each patient.

As summarized in Table 3, the rates of grade ≥ 1, grade ≥ 2, and grade ≥ 3 AHT were comparable across different chemotherapy cycles, with no statistically significant differences observed. Similarly, the incidence of grade ≥ 1, grade ≥ 2, and grade ≥ 3 AHT did not differ significantly between the 17 patients who received a local radiation boost and the 44 who did not, at all severity thresholds.

**Table 3:** Association of chemotherapy cycles and local boost with AHT

Variable	Incidence rate	*P*-value
≥1 AHT		
6 cycles	62.5%(5/8)	0.807
5 cycles	74.2%(23/31)	
4 cycles	71.4%(15/21)	
Boost	76.5%(13/17)	0.528
Non-boost	68.2(30/44)	
≥2 AHT		
6 cycles	62.5%(5/8)	0.913
5 cycles	67.7%(21/31)	
4 cycles	61.9%(13/21)	
Boost	70.6%(12/17)	0.501
Non-boost	61.4(27/44)	
≥3 AHT		
6 cycles	25.0%(2/8)	0.989
5 cycles	22.6%(7/31)	
4 cycles	23.8%(5/21)	
Boost	17.6%(3/17)	0.544
Non-boost	25.0%(11/44)	

Abbreviation: AHT, acute hematologic toxicity.

### Effects of PBM dose–volume on AHT

Dosimetric parameters for PBM were comparable between the boost and non-boost groups, with no significant differences observed. However, subgroup analysis revealed that the IBM-V40 was significantly higher in the boost group compared to the non-boost group (16.05% ± 5.77% vs 13.53% ± 4.55%; *P* = 0.038).

As shown in Table 4, patients were stratified into grade ≥ 1 AHT positive (*n* = 43) and grade ≥ 1 AHT negative (*n* = 18) groups based on the occurrence of grade ≥ 1 AHT. Dosimetric analysis revealed significantly elevated mean V_5_ (95.82 ± 2.93 vs 92.69 ± 4.13%; *P* < 0.001), V_10_ (90.24 ± 4.22 vs 85.66 ± 4.80%; *P* < 0.001), V_15_ (81.11 ± 4.74 vs 75.36 ± 5.68%; *P* < 0.001), V_20_ (70.24 ± 5.84 vs 64.38 ± 7.25%; *P* < 0.001), V_25_ (58.87 ± 6.81 vs 53.67 ± 8.36%; *P* = 0.007) and D_mean_ (28.15 ± 2.25 vs 26.43 ± 2.70 Gy; *P* = 0.007) values of the PBM in the grade ≥ 1 AHT positive group compared with the grade ≥ 1 AHT negative group.

**Table 4:** Statistics of pelvic dose–volume parameters for grade ≥ 1 AHT (positive group: *n* = 43; negative group: *n* = 18)

Parameter	PBM		IBM		LSBM		LPBM	
	Positive cohort mean (s.d.)	Negative cohort mean (s.d.)	Positive cohort mean (s.d.)	Negative cohort mean (s.d.)	Positive cohort mean (s.d.)	Negative cohort mean (s.d.)	Positive cohort mean (s.d.)	Negative cohort mean (s.d.)
V_5_ (%)	**95.82** (2.93)^***^	**92.69** **(4.13)**	99.78 (0.56)	99.12 (2.10)	99.65 (0.78)	98.41 (4.13)	**89.84** (7.79)^**^	**84.29** **(8.48)**
V_10_ (%)	**90.24** (4.22)^***^	**85.66** **(4.80)**	**97.77** (2.29)^*^	**95.54** **(4.03)**	98.41 (2.03)	96.28 (6.22)	**78.14** (10.74)^**^	**71.31** **(8.08)**
V_15_ (%)	**81.11** (4.74)^***^	**75.36** **(5.68)**	**89.36** (6.37)^**^	**84.12** **(7.07)**	96.65 (2.88)	93.77 (8.27)	**62.43** (10.68)^*^	**56.05** **(7.53)**
V_20_ (%)	**70.24** (5.84)^***^	**64.38** **(7.25)**	**72.37** (9.22)^*^	**66.38** **(8.08)**	92.85 (6.14)	90.17 (10.61)	**50.32** (11.06)^*^	**44.31** **(7.53)**
V_25_ (%)	**58.87** (6.81)^**^	**53.67** **(8.36)**	54.02 (10.67)	49.65 (8.71)	85.85 (10.87)	84.63 (12.76)	**40.31** (9.76)^*^	**34.16** **(7.29)**
V_30_ (%)	46.27 (7.95)	42.43 (8.95)	36.73 (10.22)	34.59 (9.02)	73.94 (14.42)	74.56 (14.59)	**30.32** (7.44)^**^	**24.86** **(6.86)**
V_35_ (%)	33.94 (7.59)	30.89 (8.22)	23.31 (7.75)	21.87 (7.16)	58.53 (15.46)	58.89 (16.27)	**21.54** (6.13)^**^	**17.27** **(5.74)**
V_40_ (%)	23.85 (6.98)	21.32 (6.65)	14.49 (5.13)	13.62 (4.78)	42.61 (13.59)	43.40 (16.26)	**14.20** (5.01)^*^	**11.04** **(4.09)**
V_45_ (%)	12.16 (5.18)	11.32 (4.88)	6.74 (3.14)	6.71 (2.83)	23.97 (11.73)	25.35 (14.40)	**6.41** (4.03)^*^	**4.86** **(2.35)**
D_mean_ (Gy)	**28.15** (2.25)^**^	**26.43** **(2.70)**	27.04 (2.40)	26.38 (24.20)	35.94 (3.99)	35.87 (4.88)	**22.43** (3.59)^**^	**19.96** **(2.55)**

Abbreviations: V_5_, V_10_, V_15_, V_20_, V_25_, V_30_, V_35_, V_40_, V_45,_ bone marrow volume receiving ≥5, 10, 15, 20, 25, 30, 35, 40, 45 Gy; D_mean_, mean dose; s.d., standard deviation.

Bold values indicate parameters for which a statistically significant difference was observed between the positive and negative cohorts. Statistical significance: ^*^  *P* < 0.05; ^**^  *P* < 0.01; ^***^  *P* < 0.001.

Subgroup analysis of IBM parameters demonstrated significantly higher mean V_10_ (97.77 ± 2.29 vs 95.54 ± 4.03%; *P* = 0.019), V_15_ (89.36 ± 6.37 vs 84.12 ± 7.07%; *P* = 0.003) and V_20_ (72.37 ± 9.22 vs 66.38 ± 8.08%; *P* = 0.010) values in the grade ≥ 1 AHT positive group, although no significant intergroup differences were observed in LSBM parameters. Similarly, LPBM analysis revealed statistically significant differences across all evaluated parameters, including V_5_ (89.84 ± 7.79 vs 84.29 ± 8.48%; *P* = 0.008), V_10_ (78.14 ± 10.74 vs 71.31 ± 8.08%; *P* = 0.009), V_15_ (62.43 ± 10.68 vs 56.05 ± 7.53%; *P* = 0.012), V_20_ (50.32 ± 11.06 vs 44.31 ± 7.53%; *P* = 0.020), V_25_ (40.31 ± 9.76 vs 34.16 ± 7.29%; *P* = 0.010), V_30_ (30.32 ± 7.44 vs 24.86 ± 6.86%; *P* = 0.005), V_35_ (21.54 ± 6.13 vs 17.27 ± 5.74%; *P* = 0.007), V_40_ (14.20 ± 5.01 vs 11.04 ± 4.09%; *P* = 0.011), V_45_ (6.41 ± 4.03 vs 4.86 ± 2.35%; *P* = 0.033) and D_mean_ (22.43 ± 3.59 vs 19.96 ± 2.55 Gy; *P* = 0.005).

Multivariate logistic regression analysis did not identify any dosimetric parameters as independent predictors of AHT development (all *P* > 0.05).

For patients with grade 1 AHT, clinical manifestations are typically absent and active intervention is generally unnecessary. However, grade ≥ 2 AHT is frequently associated with clinical symptoms that may compromise treatment compliance. As shown in Table 5, patients were stratified into grade ≥ 2 AHT positive (*n* = 39) and grade ≥ 2 AHT negative (*n* = 22) groups based on the occurrence of grade ≥ 2 AHT. The grade ≥ 2 AHT positive group exhibited significantly elevated mean V_5_ (96.23 ± 2.68 vs 92.54 ± 3.85%; *P* < 0.001), V_10_ (90.87 ± 3.82 vs 85.37 ± 4.49%; *P* < 0.001), V_15_ (81.74 ± 4.48 vs 75.29 ± 5.17%; *P* < 0.001), V_20_ (70.71 ± 5.91 vs 64.63 ± 6.61%; *P* < 0.001), V_25_ (59.28 ± 6.99 vs 53.88 ± 7.60%; *P* = 0.003), V_30_ (46.65 ± 8.15 vs 42.45 ± 8.27%; *P* = 0.030) and D_mean_ (28.34 ± 2.26 vs 26.41 ± 2.47 Gy; *P* = 0.001) values of the PBM compared with the grade ≥ 2 AHT negative group.

**Table 5:** Statistics of pelvic dose–volume parameters for grade ≥ 2 AHT (positive group: *n* = 39; negative group: *n* = 22)

Parameter	PBM		IBM		LSBM		LPBM	
	Positive cohort mean (s.d.)	Negative cohort mean (s.d.)	Positive cohort mean (s.d.)	Negative cohort mean (s.d.)	Positive cohort mean (s.d.)	Negative cohort mean (s.d.)	Positive cohort mean (s.d.)	Negative cohort mean (s.d.)
V_5_ (%)	**96.23** (2.68)^***^	**92.54** **(3.85)**	99.83 (0.52)	99.15 (1.92)	99.69 (0.75)	98.57 (3.76)	**90.86** (7.25)^***^	**83.49** **(8.16)**
V_10_ (%)	**90.87** (3.82)^***^	**85.37** **(4.49)**	**97.86** (2.25)^*^	**95.79** **(3.82)**	**98.58** (2.00)^*^	**96.36** **(5.63)**	**79.58** (10.07)^***^	**70.00** **(8.17)**
V_15_ (%)	**81.74** (4.48)^***^	**75.29** **(5.17)**	**89.59** (6.32)^**^	**84.66** **(7.06)**	**96.89** (2.83)^*^	**93.86** **(7.51)**	**63.73** (10.29)^***^	**54.90** **(7.40)**
V_20_ (%)	**70.71** (5.91)^***^	**64.63** **(6.61)**	**72.53** (9.17)^*^	**67.19** **(8.56)**	93.00 (6.36)	90.40 (9.66)	**51.39** (11.01)^***^	**43.50** **(7.18)**
V_25_ (%)	**59.28** (6.99)^**^	**53.88** **(7.60)**	**54.36** (11.02)^*^	**49.83** **(8.19)**	85.99 (11.32)	84.60 (11.66)	**41.06** (9.87)^**^	**33.94** **(6.79)**
V_30_ (%)	**46.65** (8.15)^*^	**42.45** **(8.27)**	37.05 (10.52)	34.42 (8.54)	74.21 (14.93)	73.97 (13.60)	**30.86** (7.49)^**^	**24.90** **(6.44)**
V_35_ (%)	34.18 (7.75)	31.02 (7.75)	23.46 (8.00)	21.86 (6.73)	58.67 (15.87)	58.59 (15.39)	**21.96** (6.14)^**^	**17.31** **(5.49)**
V_40_ (%)	23.56 (6.16)	22.30 (8.20)	14.53 (5.29)	13.71 (4.52)	42.67 (13.97)	43.15 (15.17)	**14.58** (4.99)^**^	**10.94** **(3.96)**
V_45_ (%)	12.44 (5.33)	10.98 (4.53)	6.85 (3.26)	6.53 (2.63)	24.39 (12.18)	24.36 (13.27)	**6.72** (4.08)^**^	**4.60** **(2.30)**
D_mean_ (Gy)	**28.34** (2.26)^**^	**26.41** **(2.47)**	27.10 (2.48)	26.41 (2.27)	36.17 (4.05)	35.48 (4.61)	**22.82** (3.51)^***^	**19.73** **(2.46)**

Abbreviations: V_5_, V_10_, V_15_, V_20_, V_25_, V_30_, V_35_, V_40_, V_45,_ bone marrow volume receiving ≥5, 10, 15, 20, 25, 30, 35, 40, 45 Gy; D_mean_, mean dose; s.d., standard deviation.

Bold values indicate parameters for which a statistically significant difference was observed between the positive and negative cohorts. Statistical significance: ^*^  *P* < 0.05; ^**^  *P* < 0.01; ^***^  *P* < 0.001.

In the subgroup analysis, the mean dose–volume parameters of the IBM were higher in the grade ≥ 2 AHT positive group, with V_10_ (97.86 ± 2.25 vs 95.79 ± 3.82%; *P* = 0.014), V_15_ (89.59 ± 6.32 vs 84.66 ± 7.06%; *P* = 0.003), V_20_ (72.53 ± 9.17 vs 67.19 ± 8.56%; *P* = 0.015) and V_25_ (54.36 ± 11.02 vs 49.83 ± 8.19%; *P* = 0.049) reaching statistical significance. Similarly, analysis of LSBM revealed elevated parameters in the grade ≥ 2 AHT positive group, particularly V_10_ (98.58 ± 2.00 vs 96.36 ± 5.63%; *P* = 0.043) and V_15_ (96.89 ± 2.83 vs 93.86 ± 7.51%; *P* = 0.041). For LPBM, all parameters exhibited significant differences, including V_5_ (90.86 ± 7.25 vs 83.49 ± 8.16%; *P* < 0.001), V_10_ (79.58 ± 10.07 v 70.00 ± 8.17%; *P* < 0.001), V_15_ (63.73 ± 10.29 vs 54.90 ± 7.40%; *P* < 0.001), V_20_ (51.39 ± 11.01 vs 43.50 ± 7.18%; *P* < 0.001), V_25_ (41.06 ± 9.87 vs 33.94 ± 6.79%; *P* = 0.002), V_30_ (30.86 ± 7.49 vs 24.90 ± 6.44%; *P* = 0.001), V_35_ (21.96 ± 6.14 vs 17.31 ± 5.49%; *P* = 0.002), V_40_ (14.58 ± 4.99 vs 10.94 ± 3.96%; *P* = 0.002), V_45_ (6.72 ± 4.08 vs 4.60 ± 2.30%; *P* = 0.006) and D_mean_ (22.82 ± 3.51 vs 19.73 ± 2.46 Gy; *P* < 0.001).

In multivariate logistic regression analysis, only PBM-V_15_ remained significant [odds ratio (OR), 2.653; 95% CI, 1.054–6.682; *P* = 0.038] in the final model (Table 7).

Grade ≥ 3 AHT constitutes a severe treatment-related toxicity during radiotherapy that requires stringent mitigation. As shown in Table 6, patients were stratified into grade ≥ 3 AHT positive (*n* = 14) and grade ≥ 3 AHT negative (*n* = 47) groups based on the occurrence of grade ≥ 3 AHT. The grade ≥ 3 positive group exhibited higher mean V_5_ (96.93 ± 2.52 vs 94.29 ± 3.67%; *P* = 0.007), V_10_ (91.73 ± 3.53 vs 88.04 ± 4.88%; *P* = 0.006), V_15_ (83.01 ± 3.22 vs 78.35 ± 5.79%; *P* < 0.001), V_20_ (72.45 ± 4.10 vs 67.34 ± 7.01%; *P* < 0.001), V_25_ (61.21 ± 5.91 vs 56.18 ± 7.73%; *P* = 0.014) and D_mean_ (28.66 ± 1.88 vs 27.34 ± 2.59 Gy; *P* = 0.040) values of the PBM compared with the grade ≥ 3 negative group.

**Table 6:** Statistics of pelvic dose–volume parameters for grade ≥ 3 AHT (positive group: *n* = 14; negative group: *n* = 47)

Parameter	PBM		IBM		LSBM		LPBM	
	Positive cohort mean (s.d.)	Negative cohort mean (s.d.)	Positive cohort mean (s.d.)	Negative cohort mean (s.d.)	Positive cohort mean (s.d.)	Negative cohort mean (s.d.)	Positive cohort mean (s.d.)	Negative cohort mean (s.d.)
V_5_ (%)	**96.93** (2.52)^**^	**94.29** **(3.67)**	**99.93** (0.13)^*^	**99.48** **(1.41)**	99.51 (1.07)	99.22 (2.64)	**92.43** (6.63)^*^	**86.94** **(8.43)**
V_10_ (%)	**91.73** (3.53)^**^	**88.04** **(4.88)**	97.92 (2.82)	96.87 (3.11)	98.15 (2.51)	97.67 (4.18)	**81.14** (9.24)^*^	**74.63** **(10.40)**
V_15_ (%)	**83.01** (3.22)^***^	**78.35** **(5.79)**	**91.11** (4.76)^*^	**86.83** **(7.24)**	96.55 (3.36)	95.57 (5.63)	**65.16** (8.63)^*^	**59.17** **(10.34)**
V_20_ (%)	**72.45** (4.10)^***^	**67.34** **(7.01)**	**74.71** (7.80)^*^	**69.38** **(9.37)**	94.02 (4.18)	91.47 (8.46)	**52.67** (7.92)^*^	**47.32** **(10.87)**
V_25_ (%)	**61.21** (5.91)^*^	**56.18** **(7.73)**	56.50 (9.00)	51.60 (10.43)	87.95 (6.09)	84.76 (12.48)	41.97 (6.64)	37.46 (9.99)
V_30_ (%)	48.29 (7.45)	44.19 (8.47)	38.67 (8.74)	35.34 (10.13)	75.64 (8.83)	73.67 (15.68)	**31.75** (5.02)^*^	**27.81** **(8.09)**
V_35_ (%)	35.04 (6.97)	32.44 (8.05)	24.74 (6.43)	22.34 (7.83)	58.03 (9.57)	58.82 (17.03)	**22.83** (4.52)^*^	**19.52** **(6.57)**
V_40_ (%)	23.66 (4.93)	22.94 (7.46)	14.99 (3.68)	14.00 (5.35)	41.09 (7.56)	43.37 (15.78)	14.96 (4.12)	12.76 (5.09)
V_45_ (%)	11.26 (3.12)	12.11 (5.53)	6.23 (1.29)	6.88 (3.37)	21.68 (7.37)	25.19 (13.59)	6.01 (3.36)	5.94 (3.79)
D_mean_ (Gy)	**28.66** (1.88)^*^	**27.34** **(2.59)**	27.36 (1.67)	26.70 (2.59)	36.11 (2.08)	35.87 (4.70)	22.96 (2.54)	21.33 (3.66)

Abbreviations: V_5_, V_10_, V_15_, V_20_, V_25_, V_30_, V_35_, V_40_, V_45,_ bone marrow volume receiving ≥5, 10, 15, 20, 25, 30, 35, 40, 45 Gy; D_mean_, mean dose; s.d., standard deviation.

Bold values indicate parameters for which a statistically significant difference was observed between the positive and negative cohorts. Statistical significance: ^*^  *P* < 0.05; ^**^  *P* < 0.01; ^***^  *P* < 0.001.

In the subgroup analysis, the mean dose–volume parameters of the IBM were higher in the grade ≥ 3 positive group, with V_5_ (99.93 ± 0.13 vs 99.48 ± 1.41%; *P* = 0.019), V_15_ (91.11 ± 4.76 vs 86.83 ± 7.24%; *P* = 0.021) and V_20_ (74.71 ± 7.80 vs 69.38 ± 9.37%; *P* = 0.029) reaching statistical significance. No significant intergroup differences were detected in LSBM parameters (all *P* > 0.05). For LPBM, the grade ≥ 3 positive group exhibited significantly increased mean V_5_ (92.43 ± 6.63 vs 86.94 ± 8.43%; *P* = 0.015), V_10_ (81.14 ± 9.24 vs 74.63 ± 10.40%; *P* = 0.020), V_15_ (65.16 ± 8.63 vs 59.17 ± 10.34%; *P* = 0.027), V_20_ (52.67 ± 7.92 vs 47.32 ± 10.87%; *P* = 0.046), V_30_ (31.75 ± 5.02 vs 27.81 ± 8.09%; *P* = 0.045) and V_35_ (22.83 ± 4.52 vs 19.52 ± 6.57%; *P* = 0.042) values compared with the grade ≥ 3 negative group.

**Fig. 2:**
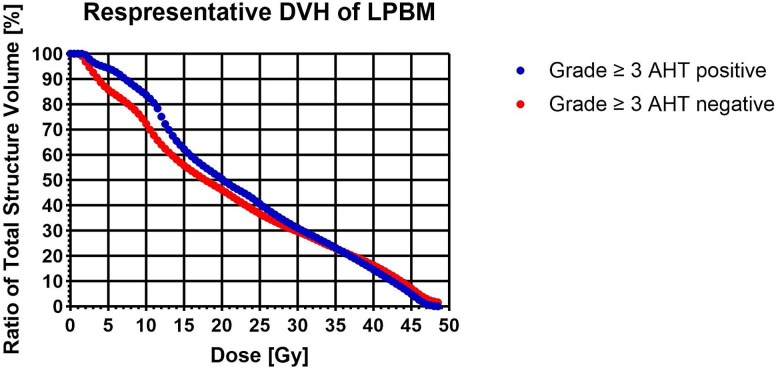
Representative dose–volume histogram (DVH) for LPBM.

As shown in Fig. 2, The representative DVH shows a gradient separating the two cohorts over the dose range of V_5_–V_20_. Multivariate logistic regression analysis identified LPBM-V_5_ as the sole independent predictor (OR, 1.425; 95% CI, 1.022–1.987; *P* = 0.037) in the final model (Table 7).

**Table 7:** Multivariate logistic regression analysis of factors associated with different AHT

Variable	Grade	β	SE	Wald	*P*	OR	95% CI
PBM-V_15_	≥2	0.976	0.471	4.289	0.038	2.653	1.054–6.682
LPBM-V_5_	≥3	0.354	0.170	4.359	0.037	1.425	1.022–1.987

Abbreviations: AHT, acute hematologic toxicity; PBM, pelvic bone marrow; LPBM, lower PBM; V_5_, V_15,_ bone marrow volume receiving ≥5, 15 Gy; β, regression coefficient; SE, standard error; OR, odds ratio; CI, confidence interval.

**Fig. 3:**
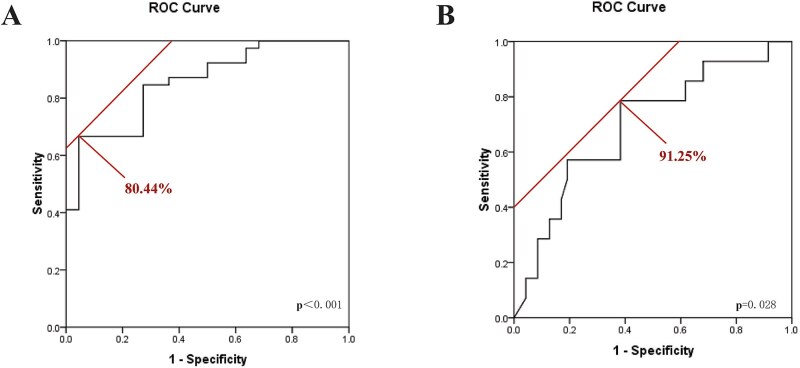
Determination of optimal cut-off value via ROC curve analysis. (A) Cutoff value of PBM under the ROC curve for Grade ≥ 2 AHT; (B) Cutoff value of LPBM under the ROC curve for Grade ≥ 3 AHT. Abbreviations: AHT, acute hematologic toxicity; PBM, pelvic bone marrow; LPBM, lower PBM; ROC, receiver operating characteristic.

As shown in Fig. 3, ROC curve analysis was conducted to evaluate the predictive performance of PBM-V_15_ for grade ≥ 2 AHT and LPBM-V_5_ for grade ≥ 3 AHT. Notably, PBM-V_15_ exhibited a significant predictive value for grade ≥ 2 AHT (AUC, 0.854; 95% CI, 0.761–0.948; *P* < 0.001), with an optimal cutoff value of 80.44% (sensitivity, 66.7%; specificity, 95.5%). Similarly, LPBM-V_5_ emerged as a moderate predictor for grade ≥ 3 AHT (AUC, 0.695; 95% CI, 0.537–0.853; *P* = 0.028), with a threshold of 91.25% (sensitivity, 78.6%; specificity, 61.7%).

## DISCUSSION

This study evaluated AHT during CCRT in a postoperative cohort of cervical and endometrial cancer patients. For cervical cancer, CCRT is standard in postoperative high-risk; for endometrial cancer, combined therapy may be considered in selected high-risk postoperative cases [4, 29]. While these malignancies exhibit distinct pathogeneses, their shared anatomical localization enables the implementation of comparable radiotherapy target volumes encompassing the pelvic lymph node region (common iliac, internal/external iliac, presacral and obturator nodal regions) [7]. As detailed in Table 8, the present dosimetric analysis revealed notable similarities in radiation dose distribution parameters between the two cancer subtypes. Although radiotherapy regimens varied among patients, all received pelvic lymph node region irradiation with a minimum dose of 45 Gy, with only 17 patients receiving an additional limited-volume local boost. Dosimetric comparison between the boost and non-boost groups revealed no significant differences in parameters except for a slightly higher IBM-V_40_ in the boost group. This difference was likely attributable to the proximity of the boosted lymph nodes to the ilium and did not affect the study’s findings regarding PBM-V_15_ and LPBM-V_5_. Critical analysis demonstrated that stringent control of pelvic bone marrow dose–volume parameters, particularly maintaining PBM-V_15_ and LPBM-V_5_ within optimal thresholds, was significantly associated with a reduced incidence of AHT during combined modality therapy. These findings highlighted the importance of bone marrow–sparing IMRT in pelvic irradiation for gynecological malignancies. The established dose–volume thresholds provide clinically actionable benchmarks to optimize therapeutic ratios in combined modality treatment approaches.

**Table 8:** Dose–volume parameters for different diseases

Parameter	PBM		IBM		LSBM		LPBM	
	Cervical cancer mean (s.d.)	Endometrial cancer mean (s.d.)	Cervical cancer mean (s.d.)	Endometrial cancer mean (s.d.)	Cervical cancer mean (s.d.)	Endometrial cancer mean (s.d.)	Cervical cancer mean (s.d.)	Endometrial cancer mean (s.d.)
V_5_ (%)	95.05 (2.89)	94.66 (4.54)	99.40 (0.67)	99.12 (1.82)	99.52 (0.89)	98.49 (4.26)	88.40 (7.31)	87.90 (9.85)
V_10_ (%)	88.75 (4.04)	89.11 (5.95)	97.32 (2.50)	96.80 (3.80)	97.82 (2.27)	97.19 (6.51)	75.63 (9.39)	76.89 (12.06)
V_15_ (%)	79.26 (4.58)	79.66 (7.08)	87.73 (6.68)	87.94 (7.51)	96.00 (3.05)	94.84 (8.66)	59.64 (8.94)	61.95 (12.00)
V_20_ (%)	68.69 (5.56)	68.25 (8.46)	70.66 (9.98)	70.50 (8.21)	92.49 (6.24)	90.65 (11.11)	47.76 (9.11)	49.75 (12.37)
V_25_ (%)	57.86 (7.06)	56.52 (8.49)	52.88 (11.38)	52.50 (8.48)	86.20 (10.97)	83.38 (13.11)	38.39 (8.66)	38.65 (10.80)
V_30_ (%)	45.92 (8.17)	43.91 (8.70)	36.29 (10.87)	35.81 (8.27)	75.21 (14.09)	70.45 (15.39)	28.90 (7.03)	28.42 (8.66)
V_35_ (%)	33.77 (7.08)	31.90 (8.29)	23.17 (8.04)	22.45 (6.87)	59.56 (14.79)	54.61 (17.12)	20.59 (6.21)	19.80 (6.51)
V_40_ (%)	23.85 (6.98)	21.96 (6.67)	14.62 (5.33)	13.63 (4.50)	43.30 (13.24)	39.99 (16.57)	13.46 (4.92)	12.97 (5.07)
V_45_ (%)	11.76 (4.66)	12.15 (5.73)	6.66 (2.93)	6.85 (3.23)	23.72 (11.15)	24.30 (15.17)	5.72 (3.35)	6.32 (4.16)
D_mean_ (Gy)	27.73 (2.25)	27.50 (2.88)	26.84 (2.54)	26.87 (2.25)	36.04 (3.72)	35.07 (5.42)	2.147 (2.92)	22.06 (4.25)

Abbreviations: PBM, pelvic bone marrow; IBM, iliac bone marrow; LSBM, lumbosacral bone marrow; LPBM, lower PBM; V_5_, V_10_, V_15_, V_20_, V_25_, V_30_, V_35_, V_40_, V_45,_ bone marrow volume receiving ≥5, 10, 15, 20, 23, 30, 35, 40, 45 Gy; D_mean_, mean dose; s.d., standard deviation.

HSPCs are radiosensitive, with mounting evidence highlighting the robust association between PBM low-dose radiation regions and AHT in pelvic radiotherapy. In a prior study by Rose *et al.* [20], elevated PBM irradiation (V_10_ ≥ 95% or V_20_ > 76%) was shown to be associated with grade ≥ 3 leukopenia in patients with cervical cancer undergoing CCRT, accompanied by strong inverse associations between nadir white blood cell counts and V_10_/V_20_ parameters in both validation and combined cohorts. Supporting this, another previous study by Mell *et al.* [19] analyzed 48 patients with anal cancer treated with CCRT and identified statistically significant associations between increased PBM-V_5_, V_10_, V_15_ and V_20_ values and reductions in leukocyte/neutrophil nadirs. Further corroborating these findings, Kumar *et al.* [30] demonstrated that adherence to constrained dosimetric thresholds (including lower pelvis V_5_ < 95%, V_20_ < 45%, iliac crest D_max_ ≤ 31 Gy and total PBM-V_20_ < 65%) effectively reduced AHT risks in 114 cervical cancer cases. These results are similar to our research findings, showing that the low-dose region (PBM-V_15_ and LPBM-V_5_) is associated with the occurrence of AHT. HSPCs exhibit extreme radiosensitivity, meaning they can be significantly damaged by relatively low radiation doses. Large-volume, low-dose irradiation can injure a substantial number of HSPCs, leading to significant depletion of the functional HSPC pool and impairment of the bone marrow’s long-term reserve and regenerative capacity. Based on these radiobiological characteristics, larger irradiation volumes cause more severe HSC damage and consequently increase the likelihood of profound bone marrow suppression. This explains our finding that the region receiving a relatively lower dose (LPBM-V_5_) correlated with Grade ≥ 3 AHT, while the region receiving a relatively higher dose (PBM-V_15_) correlated with Grade ≥ 2 AHT.

By contrast, multiple studies have emphasized the importance of limiting high-dose pelvic irradiation to reduce AHT risks. Li *et al.* [21] demonstrated that maintaining PBM-V_40_ < 25% markedly reduced the myelosuppression incidence in locally advanced cervical cancer, while Klopp *et al.* [31] and Huang *et al.* [22] identified V_40_ ≤ 37% and V_40_ < 28%, respectively, as protective thresholds against grade ≥ 2 AHT. Notably, the present cohort exhibited no significant association between high-dose irradiation (V_40_) and AHT incidence, potentially attributable to the advanced IMRT techniques employed. Through optimized dose conformity (mean V_40_ < 25%), IMRT effectively restricted excessive high-dose exposure to pelvic bone marrow, potentially counteracting the dose-dependent AHT relationships observed in conventional radiotherapy studies. This divergence highlights how modern precision radiotherapy technologies are reshaping conventional dosimetric constraint paradigms.

Current consensus contouring guidelines emphasize that delineation of pelvic lymph node region predominantly follows vascular anatomy, resulting in standardized superior pelvic target volumes due to consistent vascular topography [6, 7]. However, inferior pelvic target boundaries exhibit considerable interpatient variability, primarily driven by postoperative anatomical changes, most notably the position of the vaginal cuff. This anatomical heterogeneity translates into case-specific variations in inferior pelvic dosimetric parameters. The present subgroup analysis demonstrated no significant association between superior pelvic dose–volume metrics (including IBM and LSBM parameters) and AHT incidence, with comparable dosimetric profiles observed across groups. Notably, marked intergroup disparities emerged in inferior pelvic irradiation parameters, where V_5_ exhibited a statistically significant association with grade ≥ 3 AHT (*P* < 0.05). These findings reflect the inherent variability in target delineation, highlighting the need for dual optimization strategies: maintaining global pelvic dose constraints while implementing localized protection for inferior pelvic compartments characterized by anatomical instability. However, owing to the limited sample size, we could not systematically investigate whether specific clinical factors (e.g. vaginal invasion) quantitatively influenced LPBM parameters (e.g. V_5_) or mediated AHT risk. These findings warrant validation in larger-scale prospective cohorts to further elucidate the relationship between anatomical variations in the inferior target boundaries and radiation-induced toxicities.

Given the radiosensitivity of hematopoietic tissues, precise delineation of functional bone marrow compartments could optimize dose-sparing strategies by enhancing hematopoietic stem cell protection compared with conventional pelvic bone contouring. Although modern imaging modalities, including MRI-based techniques that differentiate marrow subtypes via fat–water composition [32, 33], as well as nuclear medicine approaches such as ^18^F-fluorodeoxyglucose positron emission tomography, ^18^F-fluorothymidine-positron emission tomography and ^99m^Tc-sulfur colloid single-photon emission computed tomography that target erythroid precursors or reticuloendothelial system macrophages [34–37], theoretically enable functional marrow mapping, these methods remain investigational due to limited clinical accessibility, high costs and technical complexity. While not employed in the current study, their potential to spatially resolve active hematopoiesis merits further exploration as an adjunct tool for personalized radiotherapy. Future work should evaluate whether integrating such advanced imaging biomarkers with conventional anatomical data could refine dose optimization without substantially escalating clinical workflows.

The management of malignant tumors involves prolonged therapeutic interventions. Patients with cervical and endometrial carcinomas often require sequential multi-cycle chemotherapy regimens following CCRT. Myelosuppression, a critical dose-limiting toxicity of chemotherapy, becomes increasingly prominent during these intensified treatment phases. This hematopoietic toxicity exerts cumulative and persistent effects on hematopoietic stem cell reserves, potentially compromising long-term hematologic recovery [38]. Preclinical investigations have demonstrated that radiation-induced damage to hematopoietic stem cells is both dose-dependent and persistent, potentially explaining the cumulative hematologic toxicity observed during sequential chemotherapy cycles in clinical practice. These observations underscore the imperative for systematic assessment of radiotherapy dosimetric parameters (particularly volumetric dose distribution). Such evaluations may critically inform therapeutic sequence optimization and survivorship management within multimodal cancer treatment protocols.

Beyond hematologic toxicity, bone marrow protection holds additional significance in the era of immunotherapy. Immunotherapy has established itself as a critical therapeutic component in gynecological oncology, showing significant clinical efficacy across multiple treatment contexts such as neoadjuvant, adjuvant, first-line and salvage therapies [39, 40]. The clinical effectiveness of immune checkpoint inhibitors exhibits a positive association with baseline peripheral lymphocyte counts and preserved immune function [41]. This association highlights the necessity of maintaining lymphocyte viability throughout multimodal treatment regimens, especially considering the marked radiosensitivity of lymphoid cells (characterized by a mean lethal dose of 1.38–2.46 Gy) [42]. Large-field pelvic radiotherapy induces substantial lymphocyte depletion through two synergistic mechanisms: (i) direct radiation-induced apoptosis of lymphocytes within the treatment field and (ii) systemic bystander effects mediated by scattered radiation doses. The cumulative lymphocyte depletion intensifies with prolonged fractionation regimens, potentially undermining the effectiveness of subsequent immunotherapy administrations. Therefore, implementing optimized radiotherapy planning strategies to reduce low-dose radiation exposure volumes combined with systematic monitoring of lymphocyte kinetics via serial CBC analyses could potentiate synergistic effects when integrating radiotherapy with immunotherapeutic approaches.

Pelvic malignancies, including colorectal and prostate cancer, represent a substantial epidemiological burden [1], with radiotherapy constituting a cornerstone treatment across disease stages. Modern radiotherapy protocols for these cancer types systematically incorporate the elective pelvic lymph node region [43, 44], inevitably increasing low-to-intermediate dose exposure to pelvic osseous structures. This common clinical scenario highlights the need for systematic investigation of pelvic bone marrow dosimetry across different cancer types. A systematic comparative dosimetric analysis of dose–volume effects in large-field pelvic irradiation cohorts could establish pan-cancer bone marrow constraints. Such evidence-based thresholds would inform the development of standardized yet customizable radiotherapy protocols that optimize the equilibrium between oncological control and hematopoietic preservation.

Current pelvic radiotherapy plan evaluations primarily focus on preventing clinically apparent toxicities in the urinary and digestive systems. Bone marrow suppression, being less symptomatic, receives comparatively less attention regarding dose constraints. However, growing evidence, including the present study, has demonstrated that restricting bone marrow irradiation reduces AHT during treatment. Our results specifically identified dose–volume parameters (PBM-V_15_, LPBM-V_5_) associated with increased hematologic toxicity risk. These findings emphasize the need to prioritize bone marrow protection in future radiotherapy planning. While current guidelines lack standardized bone marrow dose limits, the present results suggested that minimizing low-dose radiation exposure may mitigate acute bone marrow suppression, providing valuable insights for establishing future dose constraints.

The present study has several inherent limitations requiring explicit acknowledgment. First, the findings lack external validation using an independent dataset, which limits the generalizability of the proposed dose–volume thresholds (PBM-V_15_, LPBM-V_5_) and raises concerns regarding potential institutional bias. As noted in the introduction, reported dose–volume thresholds for bone marrow sparing vary considerably across institutions, reflecting differences in patient populations and treatment protocols. This heterogeneity underscores the need for validation of these constraints in diverse cohorts. Second, the recent establishment of our radiation oncology department resulted in a limited cohort size (*n* = 61), potentially reducing statistical power for subgroup comparisons. Although the results showed that PBM-V_15_ and LPBM-V_5_ had high ORs (both 95% confidence intervals >1), the calculation based on the ROC cutoff values indicated that their false negative rates were relatively high (21.4 and 33.3%, respectively), which also reflected the limitation of the limited sample size in the present study. Third, the intermediate follow-up duration precluded comprehensive assessment of long-term outcomes, including overall survival and chronic hematologic sequelae. Notwithstanding these constraints, the present preliminary findings established a crucial foundation for subsequent investigations. To enhance methodological rigor and clinical relevance, the present study is ongoing and will continue to report subsequent efficacy outcomes.

In summary, AHT during pelvic radiotherapy exhibited a significant dose–volume relationship, with low-dose irradiation parameters serving a particularly critical clinical role. The present multivariate analysis established PBM-V_15_ (threshold, 80.44%) as an independent predictor for grade ≥ 2 AHT, while LPBM-V_5_ (threshold, 91.25%) emerged as a critical determinant for grade ≥ 3 AHT.

## References

[1] Sung H, Ferlay J, Siegel RL et al. Global Cancer Statistics 2020: GLOBOCAN estimates of incidence and mortality worldwide for 36 cancers in 185 countries. CA Cancer J Clin 2021;71:209–49. 10.3322/caac.21660. 33538338

[2] Small W Jr, Bacon MA, Bajaj A et al. Cervical cancer: a global health crisis. Cancer 2017;123:2404–12. 10.1002/cncr.30667. 28464289

[3] Crosbie EJ, Kitson SJ, McAlpine JN et al. Endometrial cancer. Lancet 2022;399:1412–28. 10.1016/S0140-6736(22)00323-3. 35397864

[4] Rose PG, Bundy BN, Watkins EB et al. Concurrent cisplatin-based radiotherapy and chemotherapy for locally advanced cervical cancer. N Engl J Med 1999;340:1144–53. 10.1056/NEJM199904153401502. 10202165

[5] Wang S, Liu J, Lei K et al. Single-photon emission computed tomography-defined active bone marrow-sparing volumetric-modulated arc therapy reduces the incidence of acute hematologic toxicity in locally advanced cervical cancer patients who receive chemoradiotherapy: a single-center prospective randomized controlled trial. Cancer 2023;129:1995–2003. 10.1002/cncr.34771. 37043337

[6] Lim K, Small W, Portelance L et al. Consensus guidelines for delineation of clinical target volume for intensity-modulated pelvic radiotherapy for the definitive treatment of cervix cancer. Int J Radiat Oncol Biol Phys 2011;79:348–55. 10.1016/j.ijrobp.2009.10.075. 20472347

[7] Small W, Mell LK, Anderson P et al. Consensus guidelines for delineation of clinical target volume for intensity-modulated pelvic radiotherapy in postoperative treatment of endometrial and cervical cancer. Int J Radiat Oncol Biol Phys 2008;71:428–34. 10.1016/j.ijrobp.2007.09.042. 18037584 PMC2752724

[8] Russell WJ, Yoshinaga S, Antoku S, Mizuno M. Active bone marrow distribution in the adult. Br J Radiol 1966;39:735–9. 10.1259/0007-1285-39-466-735. 5926613

[9] Mitchell CA, Verovskaya EV, Calero-Nieto FJ et al. Stromal niche inflammation mediated by IL-1 signalling is a targetable driver of haematopoietic ageing. Nat Cell Biol 2023;25:30–41. 10.1038/s41556-022-01053-0. 36650381 PMC7614279

[10] Mauch P, Constine L, Greenberger J et al. Hematopoietic stem cell compartment: acute and late effects of radiation therapy and chemotherapy. Int J Radiat Oncol Biol Phys 1995;31:1319–39. 10.1016/0360-3016(94)00430-S. 7713791

[11] Peng QH, Chen K, Li JY et al. Analysis of treatment outcomes and prognosis after concurrent chemoradiotherapy for locally advanced cervical cancer. Front Oncol 2022;12:926840. 10.3389/fonc.2022.926840. 35992778 PMC9389882

[12] Du CH, Liu CN, Yu K et al. Mitochondrial serine catabolism safeguards maintenance of the hematopoietic stem cell pool in homeostasis and injury. Cell Stem Cell 2024;31:1484–1500.e9. 10.1016/j.stem.2024.07.009. 39181130

[13] Zhou C, Kuang M, Tao Y et al. Nynrin preserves hematopoietic stem cell function by inhibiting the mitochondrial permeability transition pore opening. Cell Stem Cell 2024;31:1359–1375.e8. 10.1016/j.stem.2024.06.007. 38955185

[14] Hu L, Yin X, Zhang Y et al. Radiation-induced bystander effects impair transplanted human hematopoietic stem cells via oxidative DNA damage. Blood 2021;137:3339–50. 10.1182/blood.2020007362. 33881475 PMC8233686

[15] Shao L, Luo Y, Zhou D. Hematopoietic stem cell injury induced by ionizing radiation. Antioxid Redox Signal 2014;20:1447–62. 10.1089/ars.2013.5635. 24124731 PMC3936513

[16] Guo CY, Luo L, Urata Y et al. Sensitivity and dose dependency of radiation-induced injury in hematopoietic stem/progenitor cells in mice. Sci Rep 2015;5:8055. 10.1038/srep08055. 25623887 PMC4306913

[17] Henry E, Arcangeli ML. How hematopoietic stem cells respond to irradiation: similarities and differences between low and high doses of ionizing radiations. Exp Hematol 2021;94:11–9. 10.1016/j.exphem.2020.12.001. 33290858

[18] Hoehn D, Pujol-Canadell M, Young EF et al. Effects of high- and low-LET radiation on human hematopoietic system reconstituted in immunodeficient mice. Radiat Res 2019;191:162–75. 10.1667/RR15148.1. 30520704 PMC6555159

[19] Mell LK, Schomas DA, Salama JK et al. Association between bone marrow dosimetric parameters and acute hematologic toxicity in anal cancer patients treated with concurrent chemotherapy and intensity-modulated radiotherapy. Int J Radiat Oncol Biol Phys 2008;70:1431–7. 10.1016/j.ijrobp.2007.08.074. 17996390

[20] Rose BS, Aydogan B, Liang Y et al. Normal tissue complication probability modeling of acute hematologic toxicity in cervical cancer patients treated with chemoradiotherapy. Int J Radiat Oncol Biol Phys 2011;79:800–7. 10.1016/j.ijrobp.2009.11.010. 20400238 PMC2907446

[21] Li W, Ma L, Li F et al. Effects of bone marrow sparing radiotherapy on acute hematologic toxicity for patients with locoregionally advanced cervical cancer: a prospective phase II randomized controlled study. Radiat Oncol 2024;19:46. 10.1186/s13014-024-02432-7. 38594678 PMC11005132

[22] Huang J, Gu F, Ji T et al. Pelvic bone marrow sparing intensity modulated radiotherapy reduces the incidence of the hematologic toxicity of patients with cervical cancer receiving concurrent chemoradiotherapy: a single-center prospective randomized controlled trial. Radiat Oncol 2020;15:180. 10.1186/s13014-020-01606-3. 32727497 PMC7389381

[23] Mell LK, Tiryaki H, Ahn KH et al. Dosimetric comparison of bone marrow-sparing intensity-modulated radiotherapy versus conventional techniques for treatment of cervical cancer. Int J Radiat Oncol Biol Phys 2008;71:1504–10. 10.1016/j.ijrobp.2008.04.046. 18640499

[24] Zhou PX, Zhang Y, Luo SG et al.. Pelvic bone marrow sparing radiotherapy for cervical cancer: a systematic review and meta-analysis. Radiother Oncol 2021;165:103–18. 10.1016/j.radonc.2021.10.015. 34718055

[25] Bhatla N, Berek JS, Cuello Fredes M et al. Revised FIGO staging for carcinoma of the cervix uteri. Int J Gynecol Obstet 2019;145:129–35. 10.1002/ijgo.12749. 30656645

[26] Berek JS, Matias-Guiu X, Creutzberg C et al. FIGO staging of endometrial cancer: 2023. Int J Gynecol Cancer 2023;33:649–66.37127326

[27] Department of Health and Human Services . Common Terminology Criteria for Adverse Events (CTCAE) v5.0. Bethesda, MD: National Cancer Institute, 2017. Available at: https://ctep.cancer.gov/protocoldevelopment/electronic_applications/ctc.htm.

[28] Collins GS, Reitsma JB, Altman DG et al.. Transparent reporting of a multivariable prediction model for individual prognosis or diagnosis (TRIPOD): the TRIPOD statement. BMJ 2014;350:g7594. 10.1136/bmj.g7594. 25569120

[29] Brooks MB, Powell ME, McCormack M et al. Adjuvant chemoradiotherapy versus radiotherapy alone in women with high-risk endometrial cancer (PORTEC-3): patterns of recurrence and post-hoc survival analysis of a randomised phase 3 trial. Lancet Oncol 2019;20:1273–85. 10.1016/S1470-2045(19)30395-X. 31345626 PMC6722042

[30] Kumar T, Schernberg A, Busato F et al. Correlation between pelvic bone marrow radiation dose and acute hematological toxicity in cervical cancer patients treated with concurrent chemoradiation. Cancer Manag Res 2019;11:6285–97. 10.2147/CMAR.S195989. 31372035 PMC6636180

[31] Klopp AH, Moughan J, Portelance L et al. Hematologic toxicity in RTOG 0418: a phase 2 study of postoperative IMRT for gynecologic cancer. Int J Radiat Oncol Biol Phys 2013;86:83–90. 10.1016/j.ijrobp.2013.01.017. 23582248 PMC4572833

[32] Hynes JP, Hughes N, Cunningham P et al. Whole-body MRI of bone marrow: a review. J Magn Reson Imaging 2019;50:1687–701. 10.1002/jmri.26759. 31016800

[33] Li X, Schwartz AV. MRI assessment of bone marrow composition in osteoporosis. Curr Osteoporos Rep 2020;18:57–66. 10.1007/s11914-020-00562-x. 31955352 PMC9044504

[34] Datz FL, Taylor A. The clinical use of radionuclide bone marrow imaging. Semin Nucl Med 1985;15:239–59. 10.1016/S0001-2998(85)80003-9. 2994233

[35] Franco P, Fiandra C, Arcadipane F et al. Incorporating ^18^FDG-PET-defined pelvic active bone marrow in the automatic treatment planning process of anal cancer patients undergoing chemo-radiation. BMC Cancer 2017;17:710. 10.1186/s12885-017-3708-4. 29096619 PMC5668955

[36] McGuire SM, Menda Y, Ponto LLB et al. 3′-deoxy-3′-[^18^F]fluorothymidine PET quantification of bone marrow response to radiation dose. Int J Radiat Oncol Biol Phys 2011;81:888–93. 10.1016/j.ijrobp.2010.12.009. 21300484 PMC3140551

[37] Wang SB, Liu JP, Lei KJ et al. The volume of ^99m^Tc sulfur colloid SPET-defined active bone marrow can predict grade 3 or higher acute hematologic toxicity in locally advanced cervical cancer patients who receive chemoradiotherapy. Cancer Med 2019;8:7219–26. 10.1002/cam4.2601. 31621208 PMC6885884

[38] Wang Y, Probin V, Zhou D. Cancer therapy-induced residual bone marrow injury: mechanisms of induction and implication for therapy. Curr Cancer Ther Rev 2006;2:271–9. 10.2174/157339406777934717. 19936034 PMC2779029

[39] Mauricio D, Zeybek B, Tymon-Rosario J et al. Immunotherapy in cervical cancer. Curr Oncol Rep 2021;23:61. 10.1007/s11912-021-01052-8. 33852056

[40] Mahdi H, Chelariu-Raicu A, Slomovitz BM. Immunotherapy in endometrial cancer. Int J Gynecol Cancer 2023;33:351–7. 10.1136/ijgc-2022-003675. 36878570 PMC11219277

[41] Peng L, Wang Y, Liu F et al. Peripheral blood markers predictive of outcome and immune-related adverse events in advanced non-small cell lung cancer treated with PD-1 inhibitors. Cancer Immunol Immunother 2020;69:1813–22. 10.1007/s00262-020-02585-w. 32350592 PMC7413896

[42] Heylmann D, Rödel F, Kindler T, Kaina B. Radiation sensitivity of human and murine peripheral blood lymphocytes, stem and progenitor cells. Biochim Biophys Acta 2014;1846:121–9. 10.1016/j.bbcan.2014.04.009. 24797212

[43] Valentini V, Gambacorta MA, Barbaro B et al. International consensus guidelines on clinical target volume delineation in rectal cancer. Radiother Oncol 2016;120:195–201. 10.1016/j.radonc.2016.07.017. 27528121

[44] Sargos P, Guerif S, Latorzeff I et al. Definition of lymph node areas for radiotherapy of prostate cancer: a critical literature review by the French Genito-Urinary Group and the French Association of Urology (GETUG-AFU). Cancer Treat Rev 2015;41:814–20. 10.1016/j.ctrv.2015.10.005. 26508669

